# The NF-*κ*B Inhibitor Curcumin Blocks Sepsis-Induced Muscle Proteolysis

**DOI:** 10.1155/2008/317851

**Published:** 2008-03-19

**Authors:** Vitaliy Poylin, Moin U. Fareed, Patrick O'Neal, Nima Alamdari, Natasha Reilly, Michael Menconi, Per-Olof Hasselgren

**Affiliations:** Department of Surgery, Beth Israel Deaconess Medical Center, Harvard Medical School, Boston, MA 02215, USA

## Abstract

We tested the hypothesis that treatment of rats with curcumin prevents sepsis-induced muscle protein degradation. In addition, we determined the influence of curcumin on different proteolytic pathways that are activated in septic muscle (i.e., ubiquitin-proteasome-, calpain-, and cathepsin L-dependent proteolysis) and examined the role of NF-*κ*B and p38/MAP kinase inactivation in curcumin-induced inhibition of muscle protein breakdown. Rats were made septic by cecal ligation and puncture or were sham-operated. Groups of rats were treated with three intraperitoneal doses (600 mg/kg) of curcumin or corresponding volumes of solvent. Protein breakdown rates were measured as release of tyrosine from incubated extensor digitorum longus muscles. Treatment with curcumin prevented sepsis-induced increase in muscle protein breakdown. Surprisingly, the upregulated expression of the ubiquitin ligases atrogin-1 and MuRF1 was not influenced by curcumin. When muscles from septic rats were treated with curcumin in vitro, proteasome-, calpain-, and cathepsin L-dependent protein breakdown rates were reduced, and nuclear NF-*κ*B/p65 expression and activity as well as levels of phosphorylated (activated) p38 were decreased. Results suggest that sepsis-induced muscle proteolysis can be blocked by curcumin and that this effect may, at least in part, be caused by inhibited NF-*κ*B and p38 activities. The results also suggest that there is not an absolute correlation between changes in muscle protein breakdown rates and changes in atrogin-1 and MuRF1 expression during treatment of muscle wasting.

## 1. INTRODUCTION

Increased breakdown of muscle proteins, in particular the myofibrillar proteins actin and myosin, is a common metabolic consequence of sepsis and other critical illness, resulting in muscle wasting, weakness, and fatigue [1, 2]. Although calpain- and cathepsin L-dependent proteolytic mechanisms are activated in atrophying muscle [3–6], ubiquitin-proteasome-dependent proteolysis plays a particularly important role in muscle wasting [7–9]. A substantial increase in the expression of the muscle-specific ubiquitin ligases atrogin-1 (MAFbx) and MuRF1 in various catabolic conditions supports the important role of the ubiquitin-proteasome pathway in loss of muscle mass [10–12].

The molecular regulation of muscle wasting is complex and involves activation of various transcription factors and nuclear cofactors regulating genes in different proteolytic pathways reviewed in [13]. Among muscle wasting-related transcription factors, NF-*κ*B has attracted much recent attention. Thus, there is evidence that NF-*κ*B is activated in skeletal muscle during sepsis [14] and in cultured myotubes treated with proinflammatory cytokines [15, 16]. Further evidence for a role of NF-*κ*B in muscle atrophy was found in a recent study in which muscle-specific overexpression of activated IkB kinase *β* **(IKK*β*) in mice resulted in phosphorylation and degradation of the inhibitory protein IkB*α*, activation of NF-*κ*B, increased proteasome-dependent protein breakdown, and muscle atrophy [17].

Curcumin (diferuloylmethane), a component of the spice turmeric (Curcuma longa) and responsible for the yellow color of curry [18, 19], possesses anti-inflammatory properties that are at least in part due to inhibition of NF-*κ*B [20, 21]. In recent studies, the effects of curcumin on the catabolic response in skeletal muscle during various muscle-wasting conditions were examined and apparently conflicting results were observed. For example, treatment of rodents with curcumin did not prevent muscle atrophy caused by muscle unloading [22], experimental cancer [23, 24], or muscular dystrophy [25]. In contrast, treatment of cultured myotubes with curcumin prevented the increase in protein degradation caused by a cachectic factor purified from experimental tumors in mice [24]. In other experiments, treatment of mice with curcumin prevented the loss of muscle weight and muscle protein caused by injection of lipopolysaccharide (LPS) [26]. Interestingly, in the same study [26], the LPS-induced upregulation of atrogin-1, but not that of MuRF1, was inhibited by curcumin.

The influence of curcumin on the catabolic response in skeletal muscle caused by sepsis is not known. In the present study, we tested the effects of curcumin on muscle proteolysis and the expression of atrogin-1 and MuRF1 in rats made septic by cecal ligation and puncture (CLP). This is a clinically relevant experimental model resembling the situation in many surgical patients with sepsis caused by intra-abdominal abscess, devitalized tissue, and a mixed aerobic and anaerobic bacterial flora in the peritoneal cavity and the blood stream [27]. We found in previous reports that CLP in rats was associated with an early activation of NF-*κ*B in skeletal muscle [14], accelerated ubiquitin-proteasome-dependent muscle proteolysis [28], and a substantial increase in atrogin-1 and MuRF1 mRNA levels [12]. In the present study, treatment of septic rats with curcumin reduced muscle protein breakdown rates and NF-*κ*B activity but, surprisingly, did not decrease the expression of atrogin-1 and MuRF1.

## 2. MATERIALS AND METHODS

### 2.1. Experimental animals

Sepsis was induced in male Sprague-Dawley rats (50–70 g) by CLP as described previously [12, 27, 28]. Other rats underwent sham-operation consisting of laparotomy and manipulation, but no ligation or puncture, of the cecum. Saline (10 mL/100 g body weight) was administered subcutaneously on the back of each rat at the time of surgery to prevent hypovolemia and septic shock. The importance of an adequate volume resuscitation to prevent hypovolemia and septic shock after CLP in rats is well documented [27, 29]. The use of 10 mL/100 g body weight is based on several previous reports from our laboratory [6, 12, 14, 28, 30] as well as unpublished observations in this laboratory showing that this volume contributes to the prevention of septic shock. Animals had free access to water, but food was withheld after the surgical procedures (both sham-operation and CLP) to avoid the influence of differences in food intake on metabolic changes between sham-operated and septic rats. Small rats were used because their lower extremity muscles are thin enough to allow for measurement of protein breakdown rates during in vitro incubation with maintained viability. The septic model used here is associated with a reproducible and substantial increase in muscle protein breakdown [12, 28, 30].

Two series of experiments were performed. In the first series of experiments, rats were treated in vivo with different amounts (described in Section 3) of curcumin (Cayman Chemical Co, Ann Arbor, Mich, USA) administered intraperitoneally 1 hour before and 8 and 15 hours after sham-operation or CLP. Control rats received corresponding volume (0.4 mL) of vehicle (0.1% DMSO in phosphate-buffered saline). 16 hours after sham-operation or CLP, extensor digitorum longus (EDL) muscles were harvested and used for determination of protein breakdown rates or mRNA and protein levels for atrogin-1 and MuRF1 as described below. When the effect of curcumin on NF-*κ*B activity was examined, muscles were studied 4 hours after CLP because we found in a previous report that the activation of NF-*κ*B in skeletal muscle was most pronounced at that time point [14].

In the second series of experiments, muscles were harvested from rats 16 hour after CLP or sham-operation and paired muscles were incubated in the absence or presence of 100 *μ*M curcumin to determine the direct effects of curcumin on protein breakdown rates, NF-*κ*B activity, p38 activation, and heat shock protein (hsp) 70 levels.

Animals were treated and cared for in accordance with National Research Council’s *Guide for the Care and Use of Laboratory Animals.* The experimental protocol was approved by the Institutional Animal Care and Utilization Committee at the Beth Israel Deaconess Medical Center.

### 2.2. Muscle incubations

Sixteen hours after CLP or sham-operation, EDL muscles were gently dissected with intact tendons, mounted on stainless steel supports at resting length, and incubated for 2 hours under physiological conditions in a shaking water bath at 37°C as described in detail previously [28, 30]. Protein breakdown rates were determined by measuring net release of free tyrosine into the incubation medium. Because tyrosine is not synthesized or degraded in muscle tissue and because reincorporation of tyrosine into protein was prevented by the presence of cycloheximide (0.5 mM) in the medium, net release of tyrosine provides a reliable measure of protein breakdown rates. In some experiments, paired muscles were incubated in absence or presence of curcumin dissolved in 0.1% DMSO (Cayman Chemical Company, Mich, USA), cathepsin L inhibitor IV (Calbiochem, EMD Biosciences, San Diego, Calif, USA), calpeptin, *β*-lactone (Boston Biochem, Cambridge, Mass, USA), the p65 inhibitory peptide PTD-p65-P1 (Imgenex, San Diego, Calif, USA), or quercetin (Sigma-Aldrich, St Louis, Miss, USA) at concentrations described in Section 3.

### 2.3. Nuclear protein isolation

Isolated nuclear proteins were used when p65 levels and p65 activity were determined. Muscle nuclei were isolated as described previously by Hunter et al. [31] with modifications. Pooled EDL muscles from 3–5 rats were homogenized in ice-cold buffer containing 10 mM HEPES (pH 7.5), 50 mM KCl, 5 mM MgCl_2_, 0.1 mM EDTA, 0.5 mM EGTA, and 0.1% Triton X-100. Samples were centrifuged at 1,000 x*g* for 5 minutes at 4°C. The supernatant (cytosolic fraction) was discarded and extraction buffer, containing 20 mM HEPES (pH 7.9), 420 mM NaCl, 25% glycerol, 0.2 mM EDTA, and 1.5 mM MgCl_2_, was added to the pellet (nuclear fraction). Samples were kept on ice for 45 minutes with vigorous vortexing every 5 minutes whereafter samples were centrifuged at 5,300 x*g* for 5 minutes at 4°C. The supernatants were applied to Amicon Ultra-4 tubes pretreated with dilution buffer containing 20 mM HEPES (pH 7.9), 40 mM KCl, 10% glycerol, 0.2 mM EDTA, and 1.5 mM MgCl_2_. After filtration, samples were centrifuged at 7,500 x*g* for 30 minutes at 4°C. Nuclear protein concentration in the supernatant was measured according to Bradford [32] using bovine serum albumin (BSA) as standard.

### 2.4. Real-time PCR

For determination of atrogin-1 and MuRF1 mRNA levels, muscle RNA was extracted and real-time PCR was performed as described in detail recently [6, 30]. The forward, reverse, and double-labeled oligonucleotides for atrogin-1 were as follows, respectively: 5′-CTT TCA ACA GAC TGG ACT TCT CGA-3′, 5′-CAG CTC CAA CAG CCT TAC TAC GT-3′, and 5′-TGC CAT CCT GGA TTC CAG AAG ATT CAA C-3′. The corresponding sequences for MuRF1 were 5′-GGA CTC CTG CCG AGT GAC C-3′, 5′-GCG TCA AAC TTG TGG CTC AG-3′, and 5′-AGG AAA ACA GCC ACC AGG TGA AGG AGG-3′. Amplification of 18S rRNA was performed in the same reaction tubes as an internal standard with an alternatively labeled probe (VIC-labeled probe) to distinguish its product from that derived from atrogin-1 and MuRF1 RNA. Atrogin-1 and MuRF1 mRNA concentrations were normalized to the 18S mRNA levels. Measurements were performed in duplicate for each standard and rat muscle sample.

### 2.5. Isolation of 20S proteasomes and measurement of proteolytic activity

Sixteen hours after CLP, paired EDL muscles were harvested and incubated for 2 hours as described above in the absence or presence of curcumin (100 *μ*M). After incubation, the muscles were rinsed in saline, blotted dry, and frozen in liquid nitrogen and stored at −80°C until analysis. To isolate 20S proteasomes, muscles were homogenized in ice-cold buffer (pH 7.5) containing 50 mM Tris-HCl, 5 mM MgCl_2_, and 250 mM sucrose. The homogenates were subjected to three sequential centrifugations. The first centrifugation was at 10,000 x*g* for 20 minutes. The supernatant was centrifuged at 100,000 x*g* for 1 hour. The supernatant from this centrifugation was centrifuged at 100,000 *g* for 5 hours. The final pellet, containing 20S proteasomes, was resuspended in buffer (pH 7.5) containing 50 mM Tris-HCl, 5 mM MgCl_2_, and 20% glycerol. Protein content of the proteasome preparation was determined according to Bradford [32] using BSA as standard. The method used here to isolate 20S proteasomes was used in a previous study from our laboratory [33]. In that study, the isolation of proteasomes was validated by electron microscopy and by demonstrating that the proteolytic activity in the proteasome fraction was blocked by proteasome inhibitors.

The activity of the 20S proteosomes was determined by measuring the cleavage of the fluorogenic substrate succinyl-leu-leu-val-tyr-7-amido-4-methylcoumarin (LLVY) (Sigma-Aldrich). This substrate is preferentially hydrolyzed by the chymotrypsin-like activity of the 20S proteasome. To measure proteolytic activity, 10 *μ*L of the 20S proteasome extract were added to 50 *μ*L of medium containing 50 mM Tris-HCl (pH 8.0), 10 mM MgCl_2_, 1 mM 1,4 dithiothreitol, 2 U Aypyrase, and 300 *μ*M LLVY. The reaction took place at 37°C for 45 minutes and was stopped by the addition of 150 *μ*L 100% cold ethanol. The peptidase activity was determined by measuring the generation of the fluorogenic cleavage product (methylcoumaryl-amide) at 380 nm excitation wavelength and 440 nm emission wavelength with a SpectraMax 5 fluorescence spectrophotometer (Molecular Devices, Union City, Calif, USA).

### 2.6. Calpain activity

To test the effect of curcumin on calpain activity, EDL muscles were harvested 16 hours after CLP and incubated in the absence or presence of curcumin (100 *μ*M). After 2 hours of incubation, muscles were rinsed in normal saline, blotted dry, frozen in liquid nitrogen, and stored at −80°C until analysis. For determination of calpain activity, frozen muscles were pulverized and homogenized in a buffer consisting of 20 mM Tris-HCl, (pH 8.0), 5 mM EDTA-Tris (pH 7.2), 0.1%** *β*-mercaptoethanol, 100 mg/L trypsin inhibitor, 2.5 *μ*M E-64, and 2 mM serine protease inhibitor phenyl-methylsulfonyl fluoride (PMSF). After centrifugation at 20,000 x*g* for 30 minutes at 4°C, protein concentration in the supernatant was determined according to Bradford [32] using BSA as standard.

Calpain activity was determined by adding aliquots of supernatant (40 *μ*g protein) to 160 *μ*L of 50 *μ*M Suc-Leu-Tyr-7-amino-4-methylcoumarin (SLY) (dissolved in DMSO and buffer consisting of 100 mM Tris-HCl and 145 mM NaCl, pH 7.3). Incubation was performed at 30°C for 30 minutes in the absence or presence of 10 mM calcium and 400 nM of z-Leu-Leu-Tyr-CHN_2_, an inhibitor of calpains and cathepsin L. Amino-4-methylcoumarin release was measured by fluorometry using 360 nm excitation and 460 nm emission filters. Calpain activity was defined as the proteolytic activity at 10 mM calcium minus the activity in the presence of calpain inhibitor and absence of calcium. Calpain activity was expressed in fluorogenic units (FU).

### 2.7. Cathepsin L activity

Sixteen hours after CLP, paired EDL muscles were incubated for 2 hours in the absence or presence of 100 *μ*M curcumin. After incubation, muscles were rinsed with saline, blotted dry, frozen in liquid nitrogen, and stored at −80°C until analysis. Muscles were homogenized in 1% Triton X-100 in PBS (pH 7.4), and the homogenates were centrifuged at 10,000 x*g* for 20 minutes at 4°C. Protein concentration in the supernatant was determined according to Bradford [32]; and aliquots (100 *μ*g protein) were used for measurement of cathepsin L activity. Cathepsin L activity was determined by using the fluorogenic peptide substrate Z-Phe-Arg-7-amido-4-methylcoumarin-HCl and the InnoZyme cathepsin L activity kit (Calbiochem) following the manufacturer’s instructions. Purified cathepsin L from human liver (Calbiochem) was used as positive control.

### 2.8. Western blotting

Aliquots (50 *μ*g total cellular protein) of muscle extracts or nuclear protein extracts were loaded on 7 × 8  cm minigels (Millipore, Badford, Mass, USA). SDS-PAGE was performed on 10% polyacrylamide gels. The separated proteins were transferred electrophoretically using semidry transfer methodology to nitrocellulose membranes (Millipore). The membranes were blocked with blocking buffer (5% nonfat dry milk, 50 mM Tris-HCl, pH 7.5, 150 mM NaCl, and 1% Tween 20) for 1 hour at room temperature. After washing with TTBS (50 mM Tris-HCl, pH 7.5, 150 mM NaCl, and 1% Tween 20) for 5 minutes × 3, membranes were incubated overnight with one of the following primary antibodies: polyclonal rabbit antihuman antiphospho p65 (Ser 536, 1:2000) (Santa Cruz Biotechnology, Santa Cruz, Calif, USA), polyclonal rabbit anti-human anti-NF-*κ*B p65 (1:2000), monoclonal mouse antihuman antiphospho p38 MAPK (Thr180/Tyr182, 1:1000), polyclonal rabbit antihuman anti-p38 MAPK (1:2000) (Cell signaling, Danvers, Mass, USA), monoclonal mouse antihuman anti-Hsp70 (1:2000), polyclonal rabbit antihuman anti-atrogin-1 (1:2000), polyclonal rabbit antihuman anti-MuRF1 (1:2000), polyclonal rabbit antihuman anti-OCT-1 (1:2000) (Santa Cruz Biotechnology), and monoclonal mouse antihuman anti-*α*-tubulin antibody (1:1000) (Sigma-Aldrich). After incubation with the primary antibodies, the membranes were washed with TTBS × 3 and incubated for 1 hour with appropriate peroxidase-conjugated secondary antibody. Membranes were then washed exhaustively in TTBS. Immunoreactive protein bands were determined by using the Western Lightning Kit for enhanced chemiluminescence (Perkin-Elmer Life Sciences, Boston, Mass, USA) and exposed on Kodak X-Omat blue film (Eastman Kodak, Rochester, NY, USA). The identity of the bands on the Western blots was confirmed by using a molecular weight ladder.

### 2.9. NF-*κ*B activity

NF-*κ*B p65 activity in the nuclear fraction was determined by using EZ-Detect NF-*κ*B Transcription Factor Kit (Pierce Biotechnology, Rockford, Ill, USA) and by following the manufacturer’s instructions. The kit utilizes streptavidin-coated 96-well plates with bound NF-*κ*B biotinylated consensus sequence and p65 binding is detected by using a p65-specific primary antibody and a secondary HRP-conjugated antibody. Wild type and mutant competitor reactions included in the kit were used to ensure signal specificity and nuclear extract from TNF*α*-activated HeLa cells was used as a positive control.

### 2.10. Statistical analysis

Results are reported as means ± SEM. Statistical analysis was performed by using Student’s *t*-test or ANOVA followed by Holm-Sidak’s or Dunn’s method as appropriate. *P* < .05 was considered statistically significant.

## 3. RESULTS

**Figure 1:**
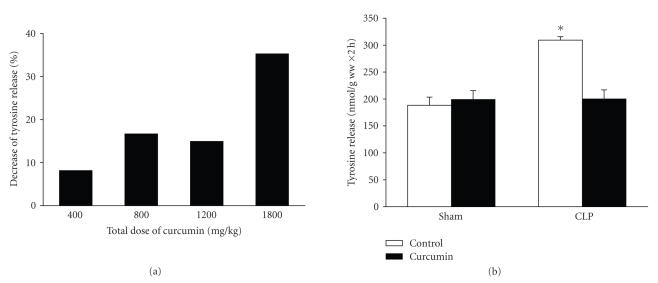
Curcumin inhibits sepsis-induced muscle proteolysis. (a) Rats were treated with different doses of curcumin and the inhibition of muscle protein breakdown in septic rats was calculated as percent inhibition of tyrosine release from muscles of curcumin-treated septic rats compared with tyrosine release from muscles of vehicle-treated septic rats. Results are from experiments in which 8 septic rats treated with vehicle and 8 septic rats treated with curcumin were studied for each total dose of curcumin indicated in the figure. (b) Sham-operated and septic rats were treated with vehicle (control) or curcumin (total dose 1800 mg/kg divided into three equal doses administered intraperitoneally 1 hour before and 8 and 15 hours after sham-operation or CLP). Protein breakdown rates were determined in incubated extensor digitorum longus muscles 16 hours after sham-operation or CLP by measuring net release of tyrosine. Results are means ± SEM with *n* = 8 in each group. *P < .05 versus all other groups by ANOVA.

In previous reports, examining the protective effects of curcumin in skeletal muscle, the dose of the drug varied substantially, ranging from 10–20 *μ*g/kg [23, 34] to 1.7 g/kg [22]. Studies suggest that doses as high as 2 g/kg can be given without toxic effects in rats [35, 36]. In initial experiments in the present study, we treated rats with three repeated doses of 60 *μ*g/kg each of curcumin administered intraperitoneally. Because no improvement in muscle protein balance was seen in those experiments, we instead used curcumin doses corresponding to the higher end of the spectrum reported previously. When rats were treated with total amounts of curcumin ranging from 400 to 1800 mg/kg divided into three doses administered intraperitonelly 1 hour before and 8 and 15 hours after sham-operation or CLP, a dose-dependent reduction of muscle protein breakdown rates was noticed in septic rats (Figure 1(a)). At the highest dose tested (three doses of 600 mg/kg), the sepsis-induced increase in muscle proteolysis was abolished (Figure 1(b)). Interestingly, the same dose of curcumin did not influence protein degradation in muscles from sham-operated rats, suggesting that curcumin specifically affected sepsis-induced muscle proteolysis without influencing basal protein breakdown.

The weight of the EDL muscle was reduced by approximately 14% in septic rats (17.8 ± 0.5 versus 20.6 ± 1.1 mg 16 hours after CLP and sham-operation, resp., means ± SEM with *n* = 8 in each group). The corresponding muscle weight in septic rats treated with three doses of 600 mg/kg of curcumin was 19.9 ± 0.9 mg, further supporting the concept that curcumin may prevent sepsis-induced muscle wasting. It should be noted, however, that none of these differences was statistically significant, most likely reflecting the fact that the present septic model is an acute model (16 hours). A more chronic model would be needed to test if curcumin can prevent sepsis-induced loss of muscle (and body) weight. Of note, in a recent study, four days of curcumin treatment prevented the loss of muscle weight induced by the injection of 1 mg/kg of LPS in mice [26].

**Figure 2:**
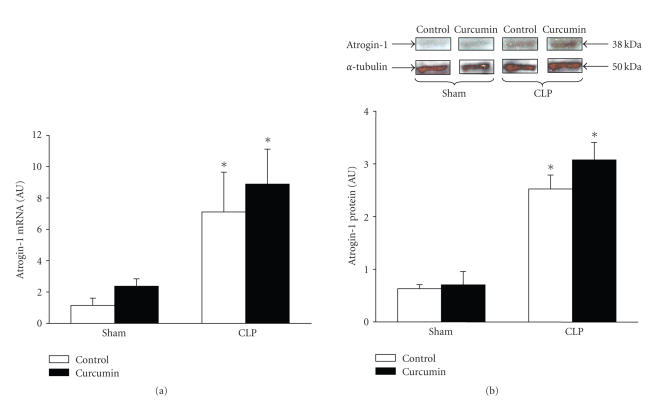
Sepsis-induced expression of atrogin-1 in extensor digitorum longus muscles is not influenced by curcumin. Sham-operated and septic rats were treated with vehicle (control) or curcumin (total dose 1800 mg/kg divided into three equal doses administered intraperitoneally 1 hour before and 8 and 15 hours after sham-operation or CLP). Muscles were harvested 16 hours after sham-operation or CLP for (a) atrogin-1 mRNA levels determined by real-time PCR and (b) atrogin-1 protein levels determined by Western blotting. In panel (b), a representative Western blot is shown in the upper portion and results from densitometric quantifications are shown in the lower portion of the figure. Results are means ± SEM with *n* = 8 in each group. AU: arbitrary units. *P < .05 versus corresponding sham group by ANOVA.

**Figure 3:**
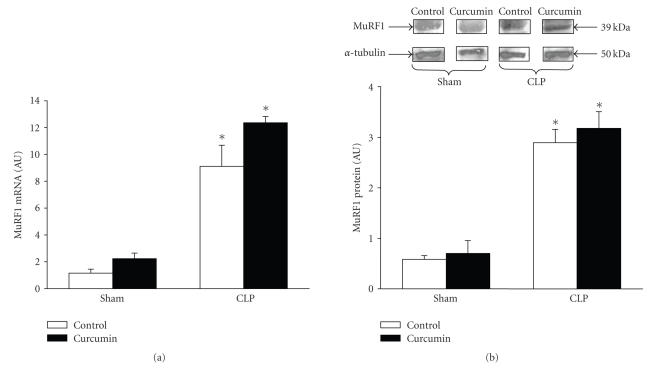
Sepsis-induced expression of MuRF1 in extensor digitorum longus muscles is not influenced by curcumin. Sham-operated and septic rats were treated with vehicle (control) or curcumin (total dose 1800 mg/kg divided into three equal doses administered intraperitoneally 1 hour before and 8 and 15 hours after sham-operation or CLP). Muscles were harvested 16 hours after sham-operation or CLP for (a) MuRF1 mRNA levels determined by real-time PCR and (b) MuRF1 protein levels determined by Western blotting. In panel (b), a representative Western blot is shown in the upper portion and results from densitometric quantifications are shown in the lower portion of the figure. Results are means ± SEM with *n* = 8 in each group. AU: arbitrary units. *P < .05 versus corresponding sham group by ANOVA.

Muscle wasting during sepsis and other catabolic conditions is typically associated with upregulated expression of several components of the ubiquitin-proetasome proteolytic pathway, in particular the ubiquitin ligases atrogin-1 and MuRF1 [10–12]. In the current study, mRNA levels for atrogin-1 and MuRF1 in muscles from septic rats were increased 7 and 9 folds, respectively, above the levels in muscles from sham-operated rats (Figures 2(a) and 3(a)). The increase in mRNA levels was accompanied by increased atrogin-1 and MuRF1 protein levels as determined by Western blotting (Figures 2(b) and 3(b)). Surprisingly, the sepsis-induced increase in atrogin-1 and MuRF1 expression was not influenced by curcumin administered at the same dose that blocked the sepsis-induced increase in muscle proteolysis (three doses of 600 mg/kg).

**Figure 4:**
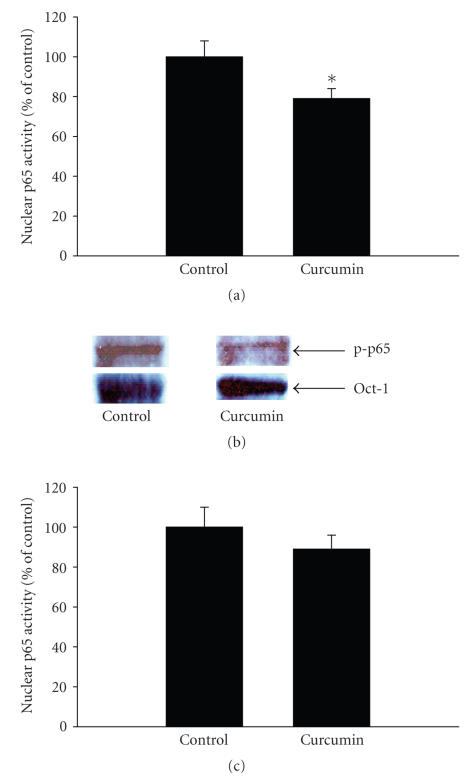
Treatment of septic rats with curcumin inhibits NF-*κ*B/p65 activity in the extensor digitorum longus muscle. Curcumin (600 mg/kg) or vehicle (control) was administered intraperitoneally 1 hour before CLP or sham-operation and muscles were harvested 4 hours later. Muscles from septic rats were used for determination of (a) p65 activity and (b) p-p65 levels in the nuclear fraction. (c) Muscles from sham-operated rats were used for measurement of p65 activity. Muscles from 2–3 rats were pooled for each measurement. Results are means ± SEM with *n* = 4 for each group and are expressed as % of control. *P < .05 versus control by Student’s *t*-test.

One of the mechanisms by which curcumin has been reported to exert anti-inflammatory and protective effects is inhibition of NF-*κ*B activity [20, 21]. We reported previously that NF-*κ*B DNA binding activity in skeletal muscle was increased after CLP in rats and that this effect of sepsis was particularly pronounced during the early phase of sepsis (4 hours after CLP) [14]. Here, we examined the effect of curcumin (600 mg/kg administered 1 hour before CLP) on NF-*κ*B activity in muscle 4 hours after CLP and found that NF-*κ*B activity, determined as p65 activity in the nuclear fraction, was reduced by approximately 30% in septic rats treated with curcumin (Figure 4(a)). The inhibitory effect on NF-*κ*B activity was further illustrated by reduced nuclear levels of phosphorylated p65 in curcumin-treated septic rats (Figure 4(b)). Previous studies provided evidence that phosphorylation of Ser 536 was associated with activation of p65 in endotoxemia [37]. In contrast, curcumin treatment of sham-operated rats did not significantly influence nuclear p65 activity (Figure 4(c)).

**Figure 5:**
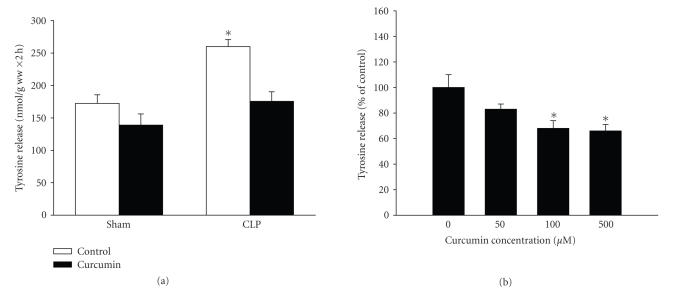
Treatment of incubated muscles in vitro with curcumin reduces sepsis-induced increase in protein breakdown. (a) Extensor digitorum longus muscles were harvested from rats 16 hours after sham-operation or CLP and incubated for 2 hours in the absence or presence of curcumin (100 *μ*M) dissolved in 0.1% DMSO. Protein breakdown rates were measured as net release of tyrosine as described in Section 2. Results are means ± SEM with *n* = 8 in each group. *P < .05 versus all other groups by ANOVA. (b) Incubated muscles from septic rats (16 hours after CLP) were treated with different concentrations of curcumin for 2 hours followed by measurement of tyrosine release. Results are means ± SEM with *n* = 8 in each group except for “0 curcumin” which was pooled from 3 paired experiments (0 versus 50, 0 versus 100, and 0 versus 500 *μ*M). Results are expressed as % of control.

Having established that treatment of rats in vivo with curcumin blocked the sepsis-induced increase in muscle proteolysis, we next examined whether the drug has a direct effect in skeletal muscle. This was done by exposing incubated muscles to curcumin in vitro. When muscles from sham-operated and septic rats were incubated in the presence of 100 *μ*M curcumin, the increased protein degradation seen in septic muscle was reduced to control levels (Figure 5(a)). This result is important because it suggests that curcumin can exert anabolic effects in catabolic muscle by a direct effect and that muscle proteolysis that has already been increased by sepsis can be reversed by curcumin. Similar to the results observed in vivo, treatment with curcumin in vitro did not result in a significant inhibition of protein breakdown rates in muscles from sham-operated rats (Figure 5). Therefore, in subsequent experiments in the present study, designed to elucidate mechanisms by which curcumin exerts its effects in catabolic muscle, we used muscles from septic rats. Because treatment of incubated muscles from septic rats with different concentrations of curcumin suggested that a maximal effect was achieved with 100 *μ*M curcumin (Figure 5(b)), this concentration was used in subsequent experiments.

Although the ubiquitin-proteasome pathway plays a significant role in muscle protein breakdown in sepsis and a number of other catabolic conditions [7–9], there is evidence that additional proteolytic mechanisms are involved. For example, previous studies suggest that calcium-calpain-dependent cleavage of myofibrillar proteins is an important “upstream” mechanism of sepsis-induced muscle proteolysis [5, 6, 38, 39]. Other studies suggest that lysosomal protein degradation, in particular cathepsin L-dependent proteolysis, is also involved in muscle wasting [3, 4].

**Figure 6:**
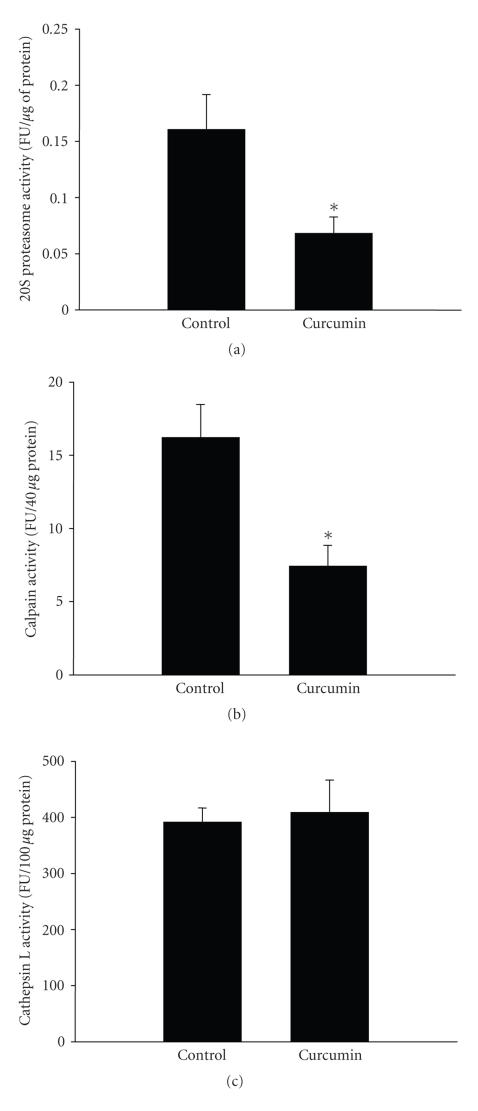
Treatment of incubated muscles from septic rats with curcumin inhibits 20S proteasome and calpain activity but does not influence cathepsin L activity. Extensor digitorum longus muscles were harvested from rats 16 hours after CLP and incubated for 2 hours in the absence or presence of curcumin (100 *μ*M) followed by measurement of (a) 20S proteasome activity, (b) calpain activity, and (c) cathepsin L activity as described in Section 2. Results are means ± SEM with *n* = 6 in each group. FU: fluorogenic units. *P < .05 versus control by Student’s *t*-test.

The influence of curcumin on individual proteolytic pathways is not known from previous studies. In order to assess the effects of curcumin on proteasomal protein degradation, incubated muscles from septic rats were treated with 100 *μ*M of the specific proteaosme inhibitor *β*-lactone [40] in the absence or presence of 100 *μ*M curcumin. Calculated as the portion of protein degradation that was blocked by *β*-lactone, the proteasome-dependent protein degradation was reduced by approximately 45% by curcumin (Table 1). In parallel experiments, treatment of incubated muscles from septic rats with curcumin reduced proteasome activity by approximately 65% (Figure 6(a)).

In order to assess the effect of curcumin on calpain-dependent protein degradation, incubated muscles from septic rats were treated with 100 *μ*M of the calpain inhibitor calpeptin [41] in the absence or presence of 100 *μ*M curcumin. The calpain-dependent protein degradation (the portion of protein degradation that was inhibited by calpeptin) was reduced by approximately 50% in the presence of curcumin (Table 1). This effect of curcumin was accompanied by a 60% inhibition of calpain activity in muscles from septic rats (Figure 6(b)).

A similar experimental approach was used to determine the influence of curcumin on cathepsin L-dependent protein degradation. When incubated septic muscles were treated with 100 *μ*M of cathepsin L inhibitor IV [42] in the presence of curcumin, the calculated cathepsin L-dependent protein degradation was reduced by approximately 40% (Table 1). In contrast, curcumin did not affect cathepsin L activity in incubated muscles from septic rats (Figure 6(c)). The reason for these apparently contradictory results with regards to curcumin-induced inhibition of cathepsin L-dependent proteolysis, but no effect on cathepsin L activity, is not known at present but may, at least in part, reflect nonspecific effects of the cathepsin L inhibitor used here [42]. Taken together, however, the results in Table 1 and Figure 6 suggest that curcumin can reduce the catabolic response to sepsis by inhibiting multiple proteolytic pathways in skeletal muscle.

**Figure 7:**
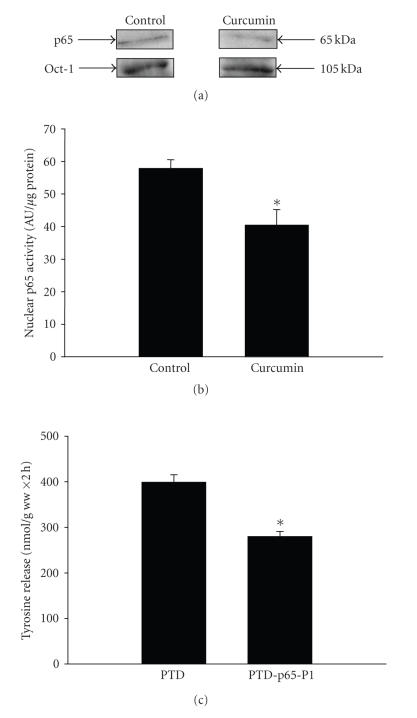
(a) p65 levels determined by Western blotting and (b) p65 activity in the nuclear fraction of extensor digitorum longus muscles from septic rats incubated for 2 hours in the absence or presence of 100 *μ*M curcumin. Results are means ± SEM with *n* = 4 in each group. AU: arbitrary units. *P < .05 versus control by Student’s *t*-test. (c) The effect of the p65 inhibitor PTD-p65-P1 on protein breakdown rates in incubated muscles from septic rats. Muscles were incubated in the presence of 100 *μ*M PTD-p65-P1 or 100 *μ*M of the inactive peptide PTD as control. Results are means ± SEM with *n* = 7 in each group. *P < .05 versus PTD by Student’s *t*-test.

Because treatment of septic rats with curcumin in vivo reduced NF-*κ*B/p65 activity in skeletal muscle (Figure 4), we tested whether a similar mechanism may be involved in the direct effect of curcumin in septic muscle. When muscles from septic rats were incubated in the presence of 100 *μ*M curcumin, the nuclear levels of p65 decreased (Figure 7(a)) and p65 DNA binding activity was reduced (Figure 7(b)). In additional experiments, treatment of incubated muscles from sham-operated rats did not significantly influence p65 activity (p65 activity was 95 ± 8% of control value in muscles from sham-operated rats treated with 100 *μ*M curcumin in vitro for 2 hours; *n* = 4 for both control and curcumin-treated muscles). The results in Figures 7(a) and 7(b) suggest that the inhibition of protein breakdown caused by treatment of incubated muscles from septic rats with curcumin may at least in part reflect inhibited NF-*κ*B/p65 activity. In order to further test the potential role of NF-*κ*B inhibition on protein breakdown, muscles from septic rats were incubated in the presence of 100 *μ*M of the specific p65 inhibitor PTD-p65-P1 [43]. This inhibitor consists of a synthetic p65 peptide containing the serine 276 phosphorylation site and linked with a protein transduction site (PTD), a short protein sequence that can enter cells to deliver its cargo without any receptor [43]. Control muscles were treated with 100 *μ*M PTD that was not linked to another peptide. Treatment of the muscles with PTD-p65-P1 resulted in an approximately 30% reduction of protein breakdown (Figure 7(c)).

**Figure 8:**
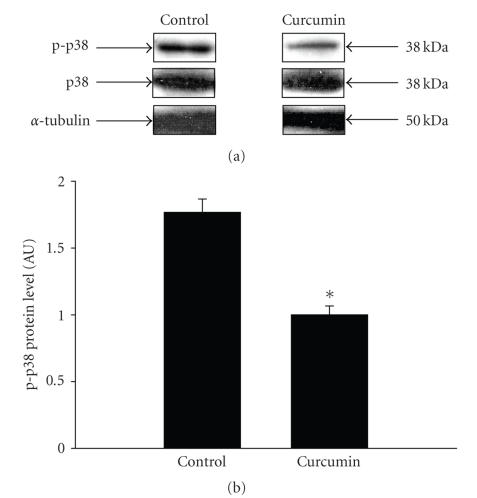
(a) Western blot analysis of phosphorylated and total p38 in septic extensor digitorum longus muscles incubated for 2 hours in the absence (control) or presence of 100 *μ*M curcumin. Muscle levels of *α*-tubulin were determined for loading control. Similar results were observed in four repeated experiments. (b) Quantification of p-p38 Western blots by densitometry. Results are means ± SEM with *n* = 4 in each group. *P < .05 versus control by Student’s *t*-test.

In addition to decreased NF-*κ*B activity, another mechanism that may be involved in the effects of curcumin is inhibition of p38 kinase activity [26, 44]. In order to test the potential role of this mechanism, the levels of phosphorylated (activated) p38 (p-p38) were determined in muscles from septic rats incubated in the presence of curcumin. Treatment of septic muscles with curcumin resulted in reduced tissue levels of p-p38, consistent with inhibited p38 activity (Figures 8(a) and 8(b)). The potential role of p38 in the regulation of muscle proteolysis was further tested by treating incubated muscles from septic rats with the p38 inhibitor SB202190. This drug specifically reduces the activity of p38*γ*, the predominant p38 isoform in skeletal muscle [45, 46]. Treatment of incubated muscles from septic rats with 50 *μ*M SB202190 resulted in an approximately 30% inhibition of protein breakdown (data not shown).

**Figure 9:**
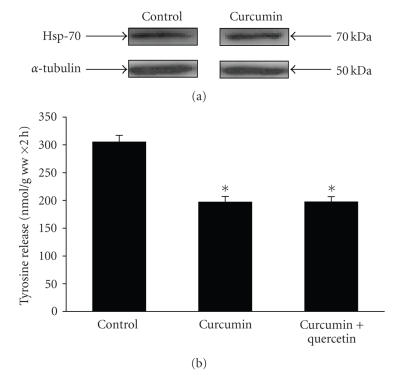
The heat shock response is not involved in the effects of curcumin on protein breakdown in incubated muscles from septic rats. (a) Muscles from septic rats (16 hours after CLP) were incubated for 2 hours in the absence (control) or presence of 100 *μ*M curcumin followed by determination of hsp-70 levels by Western blotting. Similar results were observed in three repeated experiments. (b) Protein breakdown rates in muscles from septic rats incubated for 2 hours in the absence or presence of 100 *μ*M curcumin or 100 *μ*M curcumin + 100 mM quercetin. Results are means ± SEM with *n* = 12 in each group. *P < .05 versus control by ANOVA.

In previous studies, curcumin induced the heat shock response as determined by the induction of hsp70 expression [47]. We next tested whether a similar mechanism may be involved in the effects of curcumin observed in the present experiments. When muscles from septic rats were incubated in the presence of 100 *μ*M curcumin, no changes in hsp70 levels were noticed (Figure 9(a)). In addition, the heat shock inhibitor quercetin [48] did not influence the effect of curcumin on protein degradation in muscles from septic rats (Figure 9(b)). Thus, induction of the heat shock response is probably not a major mechanism by which curcumin inhibits sepsis-induced muscle proteolysis.

## 4. DISCUSSION

In the present study, treatment of rats with curcumin blocked sepsis-induced increase in muscle proteolysis. In additional experiments, treatment of incubated muscles from septic rats with curcumin in vitro resulted in inhibited proteasomal, cathepsin L-dependent, and calpain-dependent protein degradation, suggesting that curcumin can inhibit multiple proteolytic pathways by a direct effect in catabolic muscle. The results are important because they suggest that curcumin may be useful in the prevention and treatment of muscle wasting caused by sepsis. In a recent study, curcumin prevented metabolic consequences of sepsis in the liver and prevented mortality in rats with sepsis induced by CLP [49]. The role of curcumin in the treatment of sepsis was reviewed recently by Thiemermann [50].

The dose of curcumin used in the present study was high, in particular if the dose is converted to a corresponding dose in humans (each dose of 600 mg/kg administered in rats in the present study would correspond to 42 g in a 70-kg person). It should be noted that the optimal dose of curcumin is not clear, probably reflecting differences in route of administration, length of treatment, and disease state being treated [51]. In a recent report, up to 12 g of curcumin was administered in humans without significant toxicity [52].

Because, in the present in vivo experiments, rats received the first dose of curcumin before induction of sepsis, the results may have reflected prevention, rather than treatment, of the sepsis-induced muscle proteolysis. An even more extended period of pretreatment (4 days) was used in a recent study in which curcumin prevented LPS-induced muscle wasting [26]. The results from the in vitro experiments in the present study are important because they demonstrate that muscle proteolysis that has already been activated by sepsis can be reversed by curcumin. Although any extrapolation from that observation needs to be done with caution, the result suggests that it may be possible to treat, and not only prevent, sepsis-induced muscle proteolysis. However, additional experiments, delaying curcumin therapy in vivo for several hours after induction of sepsis, will be needed to further test the role of curcumin in the treatment of sepsis-induced muscle wasting.

A pronounced increase in atrogin-1 and MuRF1 mRNA levels in skeletal muscle as observed in septic rats in the present study is similar to results in previous reports in which the expression of the ubiquitin ligases was upregulated in various catabolic conditions, including sepsis, denervation, starvation, and burn injury [10–12, 53]. Because atrogin-1 and MuRF1 mRNA levels have been suggested to be reliable molecular markers of muscle wasting, it was surprising in the current experiments that atrogin-1 and MuRF1 mRNA levels were not reduced in curcumin-treated septic rats despite inhibited muscle protein breakdown rates. A similar “disconnection” between changes in protein breakdown rates and changes in atrogin-1 and MuRF1 expression has been reported in other studies as well. For example, we found recently that treatment of rats with calpain inhibitors prevented sepsis-induced muscle proteolysis but did not influence the elevated mRNA levels for atrogin-1 and MuRF1 [30]. In other experiments, muscle-specific overexpression of active IKK*β* resulted in increased muscle proteolysis and upregulated MuRF1 expression but did not influence atrogin-1 expression [17]. In a more recent study, treatment of mice with curcumin prevented LPS-induced muscle atrophy and atrogin-1 expression but did not affect MuRF1 expression [26]. Thus, although there is a close correlation between muscle protein breakdown rates and the expression of atrogin-1 and MuRF1 in many situations, the correlation is not universal.

Only one previous study has been reported in which the effects of curcumin on the catabolic response in muscle during a sepsis-like condition were tested [26]. In that study, mice were treated for 4 days with intraperitoneal injections of curcumin (10–60 *μ*g/kg) followed by the injection of 1 mg/kg of LPS. The pretreatment with curcumin resulted in a dose-dependent inhibition of LPS-induced upregulation of atrogin-1, but not MuRF1, and loss of muscle weight and protein. The experiments reported here differ from the previous study in endotoxemic mice in several important aspects. First, CLP is a septic model that resembles the situation in many patients with abdominal sepsis and, therefore, may be clinically more relevant than a single injection of LPS. Second, whereas in the study of endotoxemic mice [26], the experimental protocol was that of curcumin pretreatment only (with no further administration of curcumin after the injection of LPS). In the present study, rats continued to be treated with curcumin during the septic course. Finally, the current experiments provided further insight into cellular mechanisms by determining the influence of curcumin treatment on individual proteolytic pathways. Despite these differences, however, both studies support the concept that the catabolic response to sepsis and endotoxemia may be prevented by curcumin.

Although curcumin can inhibit NF-*κ*B activation by preventing the phosphorylation and degradation of IkB*α* [20, 21] and is frequently used as an “NF-*κ*B inhibitor”, the drug can exert other anti-inflammatory effects as well, including inhibition of p38 kinase activity [26, 44], oxygen radical scavenging [54], and induction of the heat shock response [47]. Interestingly, the mechanisms by which curcumin provide protection may be different depending on the insult and the dose of curcumin. For example, the results in the present study suggest that curcumin (600 mg/kg administered 1 hour before CLP) prevented the early activation of NF-*κ*B in septic rats whereas 60 *μ*g/kg of the drug, administered daily to mice during 4 days before a single injection of LPS, did not inhibit NF-*κ*B DNA binding activity but instead exerted its protective effects by inhibiting p38 kinase activity [26]. It should be pointed out that although NF-*κ*B activity (determined as p65 nuclear activity) was inhibited by treatment with curcumin and the p65 inhibitor PTD-p65-P1 reduced protein breakdown in septic muscles in the present study, the results do not prove that the reduction of muscle proteolysis was caused by NF-*κ*B inhibition. Indeed, the fact that NF-*κ*B is activated early (4 hours after CLP) in the present septic model [14] and that protective effects of curcumin treatment were present up to at least 16 hours after induction of sepsis suggests that other mechanisms may have been involved as well. Results in the present study suggest that inhibition of p38 activity may be an additional mechanism by which curcumin blocked sepsis-induced muscle proteolysis, similar to the mechanism in curcumin-treated mice with endotoxemia [26]. Because, in recent experiments, we found that incubation of muscles from septic rats with the oxygen radical scavenger 2,6-di-tertbutyl-4-methylphenol did not reduce protein breakdown rates [30], it is unlikely that the effects of curcumin noted here were caused by oxygen radical scavenging. Of note, the present results do not rule out the possibility that mechanisms other than or in addition to NF-*κ*B and p38 inhibition were involved in the anticatabolic effects of curcumin.

 Despite substantial progress during the last 10–20 years in our understanding of mechanisms involved in the regulation of muscle mass, a universally accepted and effective treatment of muscle wasting is still not available. The results in the present study suggest that curcumin may be used to prevent or treat muscle catabolism, at least when caused by sepsis. This is significant because curcumin can be administered orally or parenterally at high doses without toxic effects [19, 34, 35, 51, 52, 55]. It should be noted, however, that before any clinical implications from the present results are made, several limitations of the current study need to be taken into account. First, the experiments were performed in septic rats and further studies will be needed to test if curcumin can improve protein balance in human patients with sepsis. Second, even if the present in vitro experiments suggest that changes in muscle protein breakdown that have already been induced by sepsis can be reversed by curcumin, our in vivo experiments do not allow us to conclude that curcumin can be used as treatment, rather than prevention, of muscle wasting. Finally, although the present observations suggest that the effects of curcumin on muscle proteolysis in sepsis may be related to inhibition of NF-*κ*B and p38 activity, additional studies are needed to establish the link between these mechanisms and reduced muscle proteolysis.

## 5. CONCLUSIONS

The present results suggest that curcumin can reduce sepsis-induced muscle wasting by inhibiting multiple proteolytic pathways. The finding that atrogin-1 and MuRF1 mRNA levels remained high in septic rats treated with curcumin, despite inhibited muscle protein breakdown rates, suggests that there is not an absolute correlation between changes in muscle proteolysis and changes in atrogin-1 and MuRF1 expression during treatment of muscle wasting. Although inhibition of NF-*κ*B activity is probably an important mechanism, curcumin may prevent loss of muscle mass by other mechanisms as well, including inhibition of p38 kinase activity. The observations in the present study are important because they may help develop new strategies for the prevention and treatment of muscle wasting during sepsis and other catabolic conditions.

## Figures and Tables

**Table 1:** The effects of curcumin on protein breakdown by different proteolytic pathways in incubated EDL muscles from septic rats. EDL muscles from septic rats were incubated in the absence or presence of specific proteolytic inhibitors. The inhibition of protein degradation caused by an inhibitor was calculated as the portion of protein degradation regulated by that specific proteolytic pathway. All differences caused by the proteolytic inhibitors as well as all differences induced by curcumin were statistically significant (*P* < .05) by ANOVA. Results are means ± SEM with *n* = 7 in each group.

Inhibitor	Protein degradation (nmol tyr/g ww ×2 h)		Inhibition by curcumin
	No curcumin	Curcumin	
No addition	230 ± 11	160 ± 9	
*β*-lactone (100 *μ*M)	164 ± 9	124 ± 9	
Proteasomal degradation	66	36	−45%

No addition	200 ± 13	145 ± 13	
Calpeptin (100 *μ*M)	125 ± 9	108 ± 14	
Calpain-dependent degradation	75	37	−51%

No addition	235 ± 6	171 ± 8	
Cathepsin L Inhibitor IV (100 *μ*M)	121 ± 3	106 ± 5	
Lysosomal degradation	114	65	−43%
